# Age Is Not the Limit—Functional Outcomes and Discharge Predictors in a Neurorehabilitation Cohort of Mixed Ages

**DOI:** 10.1016/j.arrct.2025.100545

**Published:** 2025-11-02

**Authors:** Mauro Silva, Armin Schnider, François Herrmann, Christophe Graf

**Affiliations:** a Division of Geriatrics and Rehabilitation, Department of Geriatrics and Rehabilitation, University Hospitals of Geneva, Geneva.; b Division of Neurorehabilitation, Department of Clinical Neurosciences, University Hospital and University of Geneva, Geneva, Switzerland.

**Keywords:** Discharge destination, Functional recovery, Neurorehabilitation, Older adults, Rehabilitation, Rehabilitation outcomes

## Abstract

**Objective:**

To identify predictors of functional improvement and home discharge across a wide age spectrum in a real-world inpatient neurorehabilitation cohort, and to assess whether age independently influences rehabilitation outcomes.

**Design:**

Retrospective observational cohort study.

**Setting:**

Two inpatient neurorehabilitation units within a university hospital: a high-intensity program (Neurorehabilitation Unit A) and a geriatric-adapted, less intensive program (Neurorehabilitation Unit B).

**Participants:**

A total of 694 patients (N=694) admitted for neurorehabilitation between January 2018 and April 2020. Mean age was 66.6±17.5 years; 47.1% were women, and 60.1% were admitted poststroke.

**Interventions:**

Not applicable.

**Main Outcome Measures:**

Home discharge; functional improvement defined as ΔFIM≥10 and Montebello Rehabilitation Factor Score (MRFS)≥0.5.

**Results:**

At discharge, 74.9% of patients returned home. Functional improvement was achieved in 32.5% (ΔFIM≥10) and 18.3% (MRFS≥0.5). In multivariable models, age was not independently associated with any outcome. Positive predictors of home discharge included higher FIM score at discharge (odds ratio, 1.05; 95% CI, 1.03-1.07), lower FIM score at admission, and greater therapy intensity. Functional improvement was associated with longer length of stay and lower comorbidity burden. Use of antipsychotics (home discharge) and antidepressants (MRFS) were negatively associated with outcomes. Hospitalization in the geriatric unit (Neurorehabilitation Unit B) was associated with lower odds of recovery and discharge home, likely reflecting increased frailty and complexity.

**Conclusions:**

Chronological age was not an independent predictor of home discharge or functional improvement in this mixed-age cohort; generalization to the oldest-old and markedly frail populations should be cautious. Functional status, comorbidities, and therapy factors were more relevant for prognosis. These findings support individualized, age-inclusive rehabilitation strategies that focus on clinical complexity rather than age alone.

Neurologic disorders are among the leading causes of disability and dependency worldwide, accounting for 11.6% of the global burden of disease and representing the second leading cause of death after cardiovascular conditions.^1^^,^^2^ Stroke alone contributes to >40% of all neurologic disability-adjusted life years, making it a key target for rehabilitation interventions.^2^ As populations age, the demand for effective inpatient neurorehabilitation programs is growing, particularly among older adults who experience significant functional decline after acute neurologic events.

Rehabilitation aims to restore independence in activities of daily living, reduce disability, and facilitate community reintegration.^3^ Evidence supports the use of structured, multidisciplinary neurorehabilitation programs in both younger and older populations,^4^^,^^5^ yet the belief persists that older adults—especially those aged ≥80 years—have limited potential for meaningful recovery.^6^, ^7^, ^8^ As a result, chronological age often influences decisions regarding access to rehabilitation, length of stay, and therapeutic intensity.

However, growing evidence suggests that age alone may not be a robust predictor of neurorehabilitation outcomes. Studies have shown that once factors such as comorbidity burden, cognitive impairment, premorbid disability, and social context are accounted for, the independent effect of age diminishes substantially.^9^^,^^10^ Furthermore, older adults may achieve comparable functional gains when given access to appropriately tailored programs.^11^^,^^12^

In this study, we retrospectively analyzed a cohort of patients admitted to 2 inpatient neurorehabilitation programs within a university hospital setting. Our primary objective was to evaluate predictors of functional improvement and home discharge across a wide age spectrum. We specifically tested the hypothesis that chronological age is not an independent determinant of neurorehabilitation outcomes when accounting for comorbidities, functional status, and therapeutic factors.

## Methods

### Design, setting, and participants

We conducted a retrospective observational cohort study using routinely collected data from a university-affiliated hospital center. The study included all patients admitted for inpatient neurorehabilitation, across 2 specialized units: Neurorehabilitation Unit A (NRA) and Neurorehabilitation Unit B (NRB). NRA primarily admitted younger patients with higher rehabilitation potential and offered a high-intensity therapy program. In contrast, NRB focused on older, polymorbid patients, offering a less intensive, geriatric-adapted program. All patients were admitted after a recent neurologic event requiring multidisciplinary rehabilitation.

In practice, NRB admission is prioritized for patients aged >75 years or for those with significant medical comorbidity, cognitive impairment, or social complexity, consistent with the demographic and clinical profiles observed in our cohort.

At NRA, therapy was typically delivered with physiotherapy sessions up to twice daily, ergotherapy once daily, and neuropsychology/logopedics once daily; nutritional consultation was provided as needed. At NRB, the regimen consisted of physiotherapy once daily, ergotherapy three times per week, neuropsychology/logopedics once daily, and systematic nutritional screening at admission with weekly follow-up.

We defined high-intensity therapy in this study as the higher frequency and cumulative session schedule used at NRA compared with NRB. We note that there is currently no universally accepted metric for rehabilitation intensity, and our operational definition is pragmatic and institution-specific.^13^

This study was approved by the Local Ethics Committee (Project ID: 2020-01955). Given its nature as a quality-of-care evaluation, the requirement for informed consent was waived for patients without documented refusal to participate in research.

### Data collection and measurements

Clinical and administrative data were extracted automatically from electronic medical records between January 1, 2018 and April 30, 2020.

As primary outcomes, we defined rate of home discharge as the percentage of patients discharged home versus the rate of institutionalization, also extracted from the medical file. The functional improvement was defined according to 2 measures:

○ **ΔFIM≥10**: representing a clinically meaningful gain in the FIM score, which ranges from 18 (total dependence) to 126 (complete independence).^14^
○ **MRFS≥0.5**: an efficiency index calculated as
  $$\text{MRFS}=\frac{\text{FIM}\;\text{discharge}-\text{FIM}\;\text{admission}}{126-\text{FIM}\;\text{admission}}$$

where values ≥0.5 indicate effective rehabilitation.^15^, ^16^, ^17^

The dataset of independent variables was composed of demographic and clinical variables, all from measurements collected routinely in clinical practice. Our study variables included age, sex, living conditions, the neurologic disorder indicating admission in a neurorehabilitation program, other associated medical conditions (hypertension, diabetes, atrial fibrillation, dyslipidemia, anxiety, depression, malnutrition, heart failure, dementia, and stroke), length of stay, number of sessions of therapy performed during hospitalization, and number of therapies per patient per 10 days. The Cumulative Illness Rating Scale (CIRS) measures the chronic medical illness (morbidity) burden while taking into consideration the severity of chronic diseases in 14 items representing individual body systems.^18^

### Statistical analysis

Continuous variables are summarized using means and standard deviations, while categorical variables are presented as absolute frequencies and percentages. Group comparisons—for example, between patients discharged home versus institutionalized, or between those with and without significant functional improvement—were conducted using Student *t* test or Kruskal-Wallis test for continuous variables, and the chi-square test for categorical variables, as appropriate.

To evaluate the independent association between age and the main outcomes (ΔFIM≥10, MRFS≥0.5, and home discharge), multivariable logistic regression models were constructed. Age was treated as a continuous variable, and all models were adjusted for key covariates, including comorbidity burden as measured by the CIRS, baseline functional status at admission, use of psychotropic medications, therapy intensity during hospitalization, and the neurorehabilitation center (NRA vs NRB) to account for center-specific effects. These models were constructed with routinely collected clinical data. The inclusion of discharge FIM score and length of stay was exploratory and may introduce overadjustment; results should therefore be interpreted with caution. A stepwise forward selection approach was applied to retain variables that significantly improved model fit. Results are reported as odds ratios (ORs) with 95% CIs and corresponding *P* values. Statistical significance was defined as a 2-sided *P* value<.05. All analyses were performed using Stata software, version 16.1 (StataCorp).^a^

## Results

### Study population

A total of 694 patients were included in the analysis, with a mean age of 66.6±17.5 years, and 47.1% were women. The primary diagnosis was stroke in 60.1% of cases (table 1). The average length of stay was 25.3±26.6 days. Regarding discharge disposition, 74.9% of patients returned home, 15.6% were institutionalized, 7.5% were transferred to another unit, and 2.7% died during hospitalization.

**Table 1:** Baseline characteristics of the neurorehabilitation cohort according to rehabilitation unit (NRA vs NRB).

Characteristics	NRA	NRB	All
N	505	189	694
Mean age ± SD (y)	61.2±16.3	81.2±11.3	66.6±17.5*
Female	226 (44.8%)	101 (53.4%)	327 (47.1%)*
Mean length of stay ± SD (d)	21.9±26.7	34.3±24.3	25.3±26.6*
Hypertension	224 (44.4%)	119 (63.3%)	343 (49.6%)*
Diabetes	84 (16.7%)	41 (21.8%)	125 (18.1%)
Atrial fibrillation	76 (15.1%)	54 (28.7%)	130 (18.8%)*
Dyslipidemia	108 (21.4%)	42 (22.3%)	150 (21.7%)
Anxiety	46 (9.1%)	21 (11.2%)	67 (9.7%)
Depression	94 (18.7%)	27 (14.4%)	121 (17.5%)
Malnutrition	35 (6.9%)	59 (31.4%)	94 (13.6%)*
Heart failure	77 (15.3%)	39 (20.7%)	116 (16.8%)
Dementia	23 (4.6%)	32 (17.0%)	55 (7.9%)*
Stroke	288 (57.0%)	129 (68.3%)	417 (60.1%)*
Mean CIRS ± SD	13.6±6.7	16.6±5.7	14.4±6.6*
Mean FIM admission ± SD	82.6±31.6	64.1±31.0	77.6±32.5*
Mean FIM discharge ± SD	92.5±28.8	68.4±33.2	85.9±31.9*
Mean ΔFIM ± SD	9.8±17.5	4.2±16.9	8.3±17.5*
ΔFIM≥10	174 (34.5%)	51 (27.1%)	225 (32.5%)
MRFS≥0.5	105 (21.2%)	20 (10.7%)	125 (18.3%)*
Home discharge	437 (86.5%)	83 (43.9%)	520 (74.9%)*
**Therapies, n; mean ± SD**			
Total number of sessions per 10d	503; 113.4±144.5	189; 15.2±19.4	692; 86.6±131.1*
**Treatment**			
Psychotropic medication	160 (33.9%)	88 (52.9%)	248 (38.5%)*
Neuroleptics	26 (5.5%)	19 (11.0%)	45 (7.0%)*
Benzodiazepines	66 (14.0%)	24 (14.0%)	90 (14.0%)
Antidepressants	68 (14.4%)	45 (26.2%)	113 (17.5%)*

NOTE. Values are expressed as mean ± SD or n (%).

⁎ Indicates statistically significant difference between groups (*P*<.05). Abbreviations: CIRS, Cumulative Illness Rating Scale; ΔFIM, change in FIM score between admission and discharge; MRFS, Montebello Rehabilitation Factor Score; NRA, Neurorehabilitation Unit A (younger/high-intensity program); NRB, Neurorehabilitation Unit B (geriatric/low-intensity program).

At admission, the mean FIM score was 77.6±32.5. By discharge, 32.5% of patients had achieved a ΔFIM≥10, and 18.3% reached an MRFS≥0.5, reflecting clinically significant functional improvement.

Baseline characteristics of the cohort stratified by rehabilitation unit (NRA vs NRB) are presented in table 1. The NRB patients were significantly older, more comorbid, and had lower functional scores at both admission and discharge than NRA patients. Outcome-related characteristics are summarized in table 2.

**Table 2:** Clinical and functional characteristics of the neurorehabilitation cohort stratified by home discharge, ΔFIM≥10, and MRFS≥0.5.

Characteristics	Home Discharge		ΔFIM		MRFS	
	No	Yes	<10	≥10	<0.5	≥0.5
N	176	520	468	225	558	125
Mean age ± SD (y)	73.8±16.3	64.2±17.3*	66.7±18.4	66.4±15.5	67.2±17.9	65.2±15.6
Female	85 (48.9%)	242 (46.5%)	223 (47.6%)	104 (46.2%)	271 (48.6%)	52 (41.6%)
Mean length of stay ± SD (d)	38.0±31.2	21.0±23.4*	18.1±20.2	40.3±31.6*	23.3±25.1	36.1±30.8*
Hypertension	95 (54.6%)	249 (47.9%)	228 (48.9%)	115 (51.1%)	275 (49.4%)	66 (52.8%)
Diabetes	33 (19.0%)	93 (17.9%)	87 (18.7%)	37 (16.4%)	104 (18.7%)	19 (15.2%)
Atrial fibrillation	44 (25.3%)	86 (16.5%)*	86 (18.5%)	44 (19.6%)	110 (19.7%)	20 (16.0%)
Dyslipidemia	38 (21.8%)	114 (21.9%)	98 (21.0%)	52 (23.1%)	124 (22.3%)	26 (20.8%)
Anxiety	20 (11.5%)	47 (9.0%)	52 (11.2%)	15 (6.7%)	56 (10.1%)	10 (8.0%)
Depression	33 (19.0%)	88 (16.9%)	80 (17.2%)	41 (18.2%)	100 (18.0%)	21 (16.8%)
Malnutrition	47 (27.0%)	47 (9.0%)*	66 (14.2%)	28 (12.4%)	86 (15.4%)	8 (6.4%)*
Heart failure	42 (24.1%)	74 (14.2%)*	86 (18.5%)	30 (13.3%)	104 (18.7%)	11 (8.8%)*
Dementia	26 (14.9%)	30 (5.8%)*	35 (7.5%)	20 (8.9%)	47 (8.4%)	8 (6.4%)
Stroke	116 (65.9%)	303 (58.3%)	277 (59.2%)	140 (62.2%)	342 (61.3%)	71 (56.8%)
Mean CIRS ± SD	17.7±6.2	13.3±6.4*	14.4±6.6	14.4±6.7	14.7±6.6	13.4±6.6*
Mean FIM admission ± SD	56.6±29.1	84.6±30.5*	83.8±33.5	64.5±25.7*	76.5±33.2	78.2±27.4
Mean FIM discharge ± SD	60.0±30.5	94.6±27.4*	82.9±34.7	92.1±24.2*	79.5±32.0	111.0±11.9*
Mean ΔFIM ± SD	3.5±16.2	9.9±17.6*	-	-	-	-
ΔFIM≥10	46 (26.4%)	179 (34.5%)	-	-	-	-
MRFS≥0.5	10 (5.8%)	115 (22.5%)*	-	-	-	-
Home discharge	-	-	340 (72.6%)	179 (79.6%)	395 (70.8%)	115 (92.0%)*
**Therapies, n; mean ± SD**	-	-				
Total number of sessions per 10 d	174; 30.2±40.6	518; 105.5±144.9	102.5±152.6	53.7±55.8	557; 94.1±142.5	125; 49.9±42.7
**Treatment**						
Psychotropic medication	101 (57.4%)	180 (34.6%)	189 (40.4%)	92 (40.9%)	238 (42.7%)	43 (34.4%)
Neuroleptics	19 (12.0%)	26 (5.3%)	34 (7.7%)	11 (5.4%)	38 (7.3%)	7 (6.0%)
Benzodiazepines	21 (13.3%)	69 (14.2%)	58 (13.2%)	32 (15.7%)	71 (13.7%)	19 (16.4%)
Antidepressants	44 (27.8%)	69 (14.2%)	75 (17.1%)	38 (18.6%)	101 (19.5%)	12 (10.3%)

NOTE. Values are expressed as mean ± SD or n (%).

⁎ Indicates statistically significant difference between comparison groups (*P*<.05). Abbreviations: CIRS, Cumulative Illness Rating Scale; ΔFIM, change in FIM score between admission and discharge; MRFS, Montebello Rehabilitation Factor Score.

### Factors associated with home discharge

In univariate analysis, patients discharged home were significantly younger (64.2±17.3y vs 73.8±16.3y, *P*<.001) and had lower comorbidity burden (CIRS score: 13.3±6.4 vs 17.7±6.2, *P*<.001). They also had fewer diagnoses of atrial fibrillation, dementia, and malnutrition and were less likely to be treated with psychotropic medications, especially antipsychotics and antidepressants.

Functionally, these patients had higher FIM scores at both admission (84.6±30.5 vs 56.6±29.1, *P*<.001) and discharge (94.6±27.4 vs 60.0±30.5, *P*<.001). They received more therapy sessions per 10 hospital days (55.5±54.3 vs 30.2±40.6, *P*<.001). Basic demographics, diagnoses, and baseline functional scores of the cohort according to home discharge are detailed in table 2.

In the multivariable logistic regression, age was not independently associated with home discharge (adjusted OR, 1.01; 95% CI, 0.99-1.03; *P*=.200). Instead, higher FIM score at discharge (OR, 1.05; 95% CI, 1.03-1.07; *P*<.001), lower FIM score at admission (OR, 0.98; 95% CI, 0.96-0.99; *P*=.011), and greater therapy intensity (OR, 1.01; 95% CI, 1.00-1.02; *P*=.006) were positively associated with home discharge. Antipsychotic use (OR, 0.53; 95% CI, 0.32-0.87; *P*=.013) and hospitalization in the geriatric unit (NRB) (OR, 0.33; 95% CI, 0.17-0.63; *P*=.001) were negatively associated with the likelihood of home discharge (table 3).

**Table 3:** Multivariable logistic regression models predicting home discharge, ΔFIM≥10, and MRFS 0.5 in the neurorehabilitation cohort.

Logistic Regression Models of Factors Associated With Discharge Home									
Univariate					Multivariate				
Predictor Variables	OR Crude	95% CI	*P*	*R*^2^	Predictor Variables	OR Adjusted	95% CI	*P*	*R*^2^
Age	0.96	(0.95-0.98)	<.001	0.056	Age	1.01	(0.99-1.03)	.200	.359
BDZ and Z-drugs	1.08	(0.64-1.84)	.762	0.000	BDZ	2.29	(1.11-4.74)	.025	
Length of stay	0.98	(0.97-0.99)	<.001	0.069	Length of stay	0.99	(0.98-1.00)	.005	
FIM admission	1.03	(1.02-1.04)	<.001	0.125	FIM admission	0.98	(0.96-0.99)	.011	
FIM discharge	1.04	(1.03-1.04)	<.001	0.193	FIM discharge	1.05	(1.03-1.07)	<.001	
Antipsychotics	0.33	(0.23-0.49)	<.001	0.048	Antipsychotics	0.53	(0.32-0.87)	.013	
Unit - NRB	0.12	(0.08-0.17)	<.001	0.165	Unit - NRB	0.33	(0.17-0.63)	.001	
Sex	1.13	(0.79-1.62)	.504	0.001	Sex	0.92	(0.58-1.46)	.722	
Therapies/10 d	1.02	(1.01-1.03)	<.001	0.160	Therapies/10 d	1.01	(1.00-1.02)	.006	
**Logistic Regression Models of Factors Associated With ΔFIM From Bellerive and Beau-Séjour**									
Anxiety	0.57	(0.31-1.03)	.062	0.004	Anxiety	0.56	(0.28-1.14)	.112	.178
CIRS	1.00	(0.98-1.02)	.959	<0.001	CIRS	0.97	(0.94-1.00)	.043	
Hypertension	1.09	(0.79-1.50)	.592	<0.001	Hypertension	1.36	(0.93-1.98)	.114	
Length of stay	1.04	(1.03-1.05)	<.001	0.126	Length of stay	1.04	(1.03-1.06)	<.001	
Unit - NRB	0.71	(0.49-1.02)	.067	0.004	Unit - NRB	0.28	(0.17-0.46)	<.001	
Therapies/10 d	0.99	(0.99-1.00)	<.001	0.037	Therapies/10 d	1.00	(0.99-1.00)	.007	
**Logistic Regression Models of Factors Associated With MRFS>0.5 From Bellerive and Beau-Séjour**									
Antidepressants	0.48	(0.25-0.90)	.022	0.010	Antidepressants	0.45	(0.23-0.91)	.026	.117
CIRS	0.97	(0.94, 1.01)	.096	0.006	CIRS	0.96	(0.93, 0.99)	.015	
Length of stay	1.02	(1.01-1.02)	<.001	0.035	Length of stay	1.02	(1.01-1.03)	<.001	
Unit - NRB	0.50	(0.30-0.85)	<.001	0.012	Unit - NRB	0.26	(0.15-0.47)	<.001	
Therapies/10 d	0.99	(0.99-1.00)	<.001	0.031	Therapies/10 d	0.99	(0.99-1.00)	<.001	

NOTE. Results are presented as odds ratios (ORs) with 95% CIs and corresponding *P* values.

Abbreviations: BDZ, benzodiazepines; CIRS, Cumulative Illness Rating Scale; ΔFIM, change in FIM score between admission and discharge; MRFS, Montebello Rehabilitation Factor Score; NRA, Neurorehabilitation Unit A; NRB, Neurorehabilitation Unit B; OR, odds ratio.

### Recovery according to the FIM instrument (ΔFIM ≥10)

Patients who achieved a ΔFIM≥10 had significantly longer hospital stays (40.3±31.6d vs 18.1±20.2d, *P*<.001), and a higher FIM score at discharge (92.1±24.2 vs 82.9±34.7, *P*<.001), despite a lower FIM score at admission (64.5±25.7 vs 83.8±33.5, *P*<.001).

There was no significant difference in age between those who recovered and those who did not (66.4±15.5y vs 66.7±18.4y, *P*=.797). Clinical characteristics and discharge outcomes stratified by ΔFIM are shown in table 2.

In the multivariable model, age was not retained as an independent predictor of functional improvement. Instead, longer length of stay (OR, 1.04; 95% CI, 1.03-1.06; *P*<.001) and lower comorbidity burden (CIRS: OR, 0.97; 95% CI, 0.94-1.00; *P*=.043) were significantly associated with ΔFIM≥10. Hospitalization in the NRB unit was inversely associated with recovery (OR, 0.28; 95% CI, 0.17-0.46; *P*<.001), and greater therapy intensity showed a weak but statistically significant negative association (OR, 1.00; 95% CI, 0.99-1.00; *P*=.007), likely reflecting that more therapies were provided to those with more severe deficits (table 3).

### Recovery according to the MRFS (MRFS>0.5)

Similar patterns were observed when using the MRFS. Patients with MRFS≥0.5 had longer hospitalizations (36.1±30.8d vs 23.3±25.1d, *P*<.001), higher discharge FIM score (111.0±11.9 vs 79.5±32.0, *P*<.001), and lower comorbidity scores (CIRS: 13.4±6.6 vs 14.7±6.6; *P*=.030). Malnutrition and heart failure were more prevalent in the nonrecovered group (*P*=.008 for both). Clinical characteristics and discharge outcomes stratified by the MRFS are shown in table 2. Again, there was no significant difference in age between recovered and nonrecovered patients (65.2±15.6y vs 67.2±17.9y, *P*=.212).

In multivariable analysis, age was not independently associated with MRFS recovery. Instead, each additional day of hospitalization increased the odds of MRFS≥0.5 by 2% (OR, 1.02; 95% CI, 1.01-1.03; *P*<.001), and higher comorbidity burden (OR, 0.96; 95% CI, 0.93-0.99; *P*=.015) and antidepressant use (OR, 0.45; 95% CI, 0.23-0.91; *P*=.026) were negatively associated with recovery. Greater therapy intensity again showed a negative association (OR, 0.99; 95% CI, 0.99-1.00; *P*<.001) (table 3).

## Discussion

In this retrospective cohort study of nearly 700 patients admitted to 2 inpatient neurorehabilitation programs, we found that chronological age was not an independent predictor of functional improvement after adjustment for clinical and functional covariates. Although older age appeared to be associated with less favorable outcomes in univariate analysis, this effect was no longer significant in multivariable models that included comorbidity burden, functional status, therapy intensity, and psychotropic medication use. These findings challenge the assumption that age inherently limits rehabilitation potential. We note, however, that our regression models included variables such as discharge FIM score and length of stay, which may represent potential overadjustment. These results should therefore be interpreted as exploratory. Age has long been debated as either a limiting factor^7^^,^^19^ or a nonsignificant one^9^^,^^20^ in functional improvement. Supporting the latter, Khoo et al^11^ compared patients aged >65 and <65 years and found no significant difference in length of stay. Interestingly, older patients demonstrated greater functional gains and rehabilitation efficiency than their younger counterparts, reinforcing the notion that older adults can meaningfully benefit from inpatient rehabilitation when appropriately supported. However, given the cohort’s mean age and likely underrepresentation of the subgroup aged ≥85 years, our findings should be interpreted cautiously in the oldest-old and in patients with advanced frailty, particularly as no dedicated frailty scale was available in our dataset.

Neuroplasticity and motor learning capacity represent key mechanisms underlying functional improvement after neurologic injury. Although advancing age is associated with reduced cortical excitability, slower synaptogenesis, and less efficient neurotransmission, neuroplasticity is not abolished in older adults. Experimental and clinical evidence demonstrates that appropriately structured, task-specific, and cognitively engaging rehabilitation can still elicit meaningful neural reorganization and behavioral gains. Conversely, coexisting cognitive impairment or neurodegenerative disease may further attenuate neuroplastic responses and limit recovery potential.^21^, ^22^, ^23^ Our findings, showing that age per se was not an independent predictor of outcome, are consistent with the persistence of adaptive plasticity across the adult lifespan when adequate therapeutic stimulation is provided.

Although age was not an independent predictor of home discharge in our adjusted models, several important nonclinical factors likely influence this outcome. In the local context, patients benefit from a robust home care system capable of providing up to 4 daily visits, including assistance with personal care, medication management, household tasks, and therapeutic support. Nonetheless, access to such services may not fully compensate for the absence of informal caregivers. Social and familial support remains a decisive determinant of discharge destination: the presence of a spouse or close family member often facilitates home discharge, whereas its absence can necessitate institutionalization, even in patients with adequate functional improvement. This type of support is more commonly available to younger patients and has been identified as a key facilitator of home discharge.^24^ These contextual factors may help explain the observed disparities in discharge disposition across age groups and reinforce the importance of integrating social determinants into rehabilitation planning.

Our results align with earlier studies suggesting that functional improvement is more strongly influenced by premorbid health status and comorbidities than by age itself.^10^^,^^25^ As observed in prior studies,^9^^,^^11^^,^^12^^,^^26^ older adults—including those >80 years—can achieve meaningful gains in independence when provided with appropriately adapted rehabilitation programs. In our cohort, functional improvement (both ΔFIM≥0 and MRFS≥0.5) was observed across age groups, with no statistically significant differences in age between recovered and nonrecovered patients.

The strongest predictors of successful outcomes were lower comorbidity burden, better functional status at discharge, and, for home discharge, lower functional status at admission. Lower comorbidity burden was a consistent predictor of better outcomes: each additional point in the CIRS was associated with a 3%-4% reduction in the likelihood of functional improvement (ΔFIM≥10: OR, 0.97; 95% CI, 0.94-1.00; MRFS≥0.5: OR, 0.96; 95% CI, 0.93-0.99). These findings support the concept that a lower initial FIM score may offer greater potential for functional gain, as patients may be in the early subacute phase of neurologic recovery. This is in line with the idea that functional reserve and recovery trajectory are more important than age per se.

Therapy intensity demonstrated different associations across outcomes. Patients discharged home received more therapy sessions, consistent with their higher functional potential and shorter hospital stays. However, therapy intensity was not independently related to the magnitude of functional improvement (ΔFIM or MRFS) once comorbidities and baseline function were considered. This likely reflects confounding by indication, whereby patients with greater initial impairment received more intensive therapy but still achieved slower or incomplete recovery—a phenomenon consistently reported in other rehabilitation cohorts.^27^, ^28^, ^29^

We also observed that psychotropic medication use—particularly antipsychotics and antidepressants—was negatively associated with both functional outcomes and home discharge. This may reflect underlying behavioral or neuropsychiatric disturbances (eg, poststroke depression, agitation, or apathy) that limit participation in therapy.^30^^,^^31^ Whether the medications themselves, the underlying indications, or both contribute to this association remains an important question for future prospective research.

Importantly, hospitalization in the geriatric neurorehabilitation unit (NRB) was associated with lower odds of home discharge and functional improvement. This association likely reflects the objectively higher comorbidity burden, older age, and greater prevalence of conditions such as dementia and malnutrition observed in NRB patients (table 1)—characteristics consistent with a frailty-related clinical profile, although frailty was not formally measured. These differences likely reflect the higher comorbidity burden, frailty, and social complexity of NRB patients rather than differences in care quality between units. Both programs operate within the same multidisciplinary framework and institutional standards, but NRB is specifically designed to meet the needs of more medically and functionally complex patients. The fact that many older adults nonetheless achieved meaningful recovery reinforces the relevance and value of tailored geriatric rehabilitation programs.

### Study limitations

Strengths of this study include its large sample size, standardized data collection from routine clinical care, and the comparison of 2 distinct but complementary rehabilitation models within the same health care system. However, several limitations must be acknowledged. First, the retrospective design limits our ability to infer causality. Second, certain potentially relevant factors, such as cognitive status, mood assessments, caregiver support, and premorbid function, were not available in the dataset and could have influenced outcomes. Moreover, we did not capture a standardized frailty measure (eg, Clinical Frailty Scale). As a result, the external validity of our age-related findings to very old and highly frail populations is limited, and residual confounding by frailty cannot be excluded. Third, our population was drawn from a single institution, which may limit generalizability. Fourth, contextual variables such as social and familial support (eg, presence of a spouse or adult children) were not captured in the dataset, even though they likely influence discharge planning. Similarly, we lacked standardized measures of cognitive impairment and mood severity, despite their well-established impact on rehabilitation potential and discharge disposition. As such, our models may not fully account for these unmeasured domains, and residual confounding cannot be excluded. Future studies should integrate these assessments to provide a more comprehensive prognostic model. In addition, our modeling approach has inherent constraints. We did not test nonlinear relationships (eg, splines) for continuous predictors, and we analyzed ΔFIM and MRFS only as dichotomous outcomes, which may obscure finer gradations of recovery. Moreover, our simplification of discharge destination into ‘home versus institution’ does not capture the heterogeneity of posthospital trajectories such as nursing home placement, transfer to another unit, or in-hospital death. These aspects should be addressed in future research.

Finally, we acknowledge that our use of stepwise regression for model construction has inherent limitations. Stepwise approaches are known to be unstable, may overfit the data, and rely heavily on statistical significance rather than clinical reasoning. As such, our findings should be considered exploratory and interpreted with caution. Future studies should apply more robust approaches, including clinically driven models, penalized regression methods, such as least absolute shrinkage and selection operator, and internal validation techniques (eg, bootstrapping), to enhance model stability and reproducibility.

### Implications for practice and policy

Our findings have clear implications for rehabilitation triage and policy. Older adults should not be excluded from inpatient rehabilitation solely based on age. Instead, comprehensive assessment of functional status, comorbidities, and recovery potential should guide decision making. Age-inclusive rehabilitation models, particularly those integrating geriatric expertise, are essential to ensure equitable access and optimize outcomes for the growing population of older adults with neurologic disorders. In these patients, therapy should be tailored to individual tolerance, favoring shorter but more frequent sessions while maintaining sufficient overall therapy time, and should focus on task-specific, functionally meaningful activities that promote independence and participation in daily life.

## Conclusions

In this large retrospective cohort study, chronological age was not an independent predictor of functional improvement or home discharge (mean cohort age 66.6y). However, these findings should be interpreted with caution in the oldest-old and markedly frail populations, for whom further validation is needed. After adjusting for comorbidities, baseline functional status, and therapeutic factors, the effect of age was no longer significant. Overall, our results support the view that rehabilitation decisions should be guided by a comprehensive assessment of health status, recovery potential, and care environment rather than by age alone.

Older adults, including those with high comorbidity burdens, can achieve meaningful functional gains when offered tailored rehabilitation. By ‘tailored rehabilitation,’ we refer to programs that integrate a comprehensive geriatric assessment, multidisciplinary coordination, and caregiver involvement; adapt therapy frequency and duration to patient tolerance, often through shorter, more frequent sessions that minimize fatigue while maintaining adequate weekly therapy time; and emphasize task-specific, ecologically valid interventions that promote meaningful independence and an acceptable quality of life for both the patient and their family. The principle of ecological validity, ensuring that therapeutic gains translate effectively into real-life functioning, is central to geriatric and neurorehabilitation practice.

Equitable access to neurorehabilitation should therefore be guided by functional need and capacity for improvement, not by age-based assumptions. Future research should further explore the role of contextual factors, such as social support and postdischarge services, to better personalize rehabilitation strategies and promote successful reintegration into the community across all age groups.

## Supplier

a. Stata, version 16.1; StataCorp.
